# Phase 1 study of the ATR inhibitor berzosertib in combination with cisplatin in patients with advanced solid tumours

**DOI:** 10.1038/s41416-021-01406-w

**Published:** 2021-05-26

**Authors:** Geoffrey I. Shapiro, Robert Wesolowski, Craig Devoe, Simon Lord, John Pollard, Bart S. Hendriks, Martin Falk, Ivan Diaz-Padilla, Ruth Plummer, Timothy A. Yap

**Affiliations:** 1 Department of Medical Oncology, Dana-Farber Cancer Institute and Harvard Medical School, Boston, MA USA; 2 Medical Oncology, The Ohio State University Comprehensive Cancer Center, Columbus, OH USA; 3 Medical Oncology and Hematology, Zucker School of Medicine at Hofstra/Northwell, New York, NY USA; 4 Department of Oncology, University of Oxford, Oxford, UK; 5 Biological Sciences, Vertex Pharmaceuticals Europe Ltd, Abingdon, UK; 6 Clinical Pharmacology, EMD Serono Research & Development Institute, Inc., Billerica, MA, USA, an affiliate of Merck KGaA, Darmstadt, Germany; 7 Oncology Global Clinical Development, Merck KGaA, Darmstadt, Germany; 8 Oncology Global Clinical Development, Ares Trading SA, Eysins, Switzerland, an affiliate of Merck KGaA, Darmstadt, Germany; 9 Translational and Clinical Research Institute, Newcastle University and Northern Centre for Cancer Care, Newcastle Hospitals NHS Trust, Newcastle Upon Tyne, UK; 10 Drug Development Unit, Royal Marsden Hospital and The Institute of Cancer Research, London, UK; 11 Present Address: Bayer plc, Reading, UK; 12 Present Address: Novartis Institutes for BioMedical Research, Cambridge, MA USA; 13 Present Address: CureVac, Tübingen, Germany; 14 Present Address: GlaxoSmithKline, Zug, Switzerland; 15 Present Address: University of Texas MD Anderson Cancer Center, Houston, TX USA

**Keywords:** Medical research, Cancer

## Abstract

**Background:**

Berzosertib (formerly M6620, VX-970) is a highly potent and selective, first-in-class ataxia telangiectasia-mutated and Rad3-related protein kinase (ATR) inhibitor. We assessed the safety, tolerability, pharmacokinetics, and preliminary efficacy of berzosertib plus cisplatin.

**Methods:**

Adult patients with advanced solid tumours refractory or resistant to standard of care therapies received ascending doses of cisplatin (day 1) and berzosertib (days 2 and 9) every 3 weeks (Q3W).

**Results:**

Thirty-one patients received berzosertib (90–210 mg/m^2^) and cisplatin (40–75 mg/m^2^) across seven dose levels. The most common grade ≥3 treatment-emergent adverse events were neutropenia (20.0%) and anaemia (16.7%). There were two dose-limiting toxicities: a grade 3 hypersensitivity reaction and a grade 3 increase in alanine aminotransferase. Berzosertib 140 mg/m^2^ (days 2 and 9) and cisplatin 75 mg/m^2^ (day 1) Q3W was determined as the recommended Phase 2 dose. Cisplatin had no apparent effect on berzosertib pharmacokinetics. Of the 31 patients, four achieved a partial response (two confirmed and two unconfirmed) despite having previously experienced disease progression following platinum-based chemotherapy.

**Conclusions:**

Berzosertib plus cisplatin is well tolerated and shows preliminary clinical activity in patients with advanced solid tumours, warranting further evaluation in a Phase 2 setting.

**Clinical Trials Identifier:**

NCT02157792.

## Background

The standard of care therapies for the treatment of many tumour types include DNA-damaging chemotherapy agents.^1^ Although chemotherapy may be initially effective, the development of drug resistance is common.^2^ There is an unmet need across different cancers for novel treatments that overcome chemotherapy resistance and improve clinical outcomes.

Ataxia-telangiectasia-mutated (ATM) and Rad3-related protein kinases (ATR) play critical roles in the DNA-damage response (DDR), and regulate cell cycle checkpoint control and the repair of damaged DNA by homologous recombination.^3^ To mediate replication fork stabilisation, ATR is recruited to regions of exposed single-stranded DNA, commonly formed at stalled replication forks resulting from replication stress. ATM is recruited to DNA double-strand breaks that can result from collapsed forks.^4^

Many chemotherapy drugs, including cisplatin, work by inducing potentially lethal DNA damage in cancer cells, although their efficacy is compromised by the efficient repair of DNA damage mediated through the DDR pathway.^5^ Cancer cells harbouring ATM-p53 signalling pathway defects are increasingly reliant on ATR to mitigate DNA damage.^6^ Consistently, preclinical data show a potential lethal sensitivity to ATR inhibition in the presence of DNA damage induced by chemotherapeutic agents, which can ultimately lead to cell death via synthetic lethality.^7–10^ In contrast, non-cancer cells can tolerate the effects of ATR inhibition as a result of intact compensatory ATM signalling. Consequently, ATR inhibition may reduce the DDR capabilities of cancer cells, thereby sensitising them to chemotherapy-induced DNA damage.^11^

Berzosertib (formerly M6620, VX-970) is an intravenous (i.v.), highly potent (IC_50_ = 19 nM), and selective, first-in-class ATR inhibitor.^12^ Preclinical studies of berzosertib have shown synergy in combination with cisplatin, which led to anti-tumour responses in patient-derived lung cancer xenografts that were otherwise insensitive to cisplatin monotherapy.^12^ The timing of berzosertib administration relative to chemotherapy is critical as preclinical investigations have shown that optimal efficacy is achieved by administering berzosertib 12–24 h after cisplatin.^13^

The purpose of this first-in-human, open-label, Phase 1 trial (ClinicalTrials.gov, identifier: NCT02157792) was to evaluate the safety, tolerability, pharmacokinetics (PKs), and preliminary anti-tumour activity of berzosertib in combination with cisplatin.

## Methods

### Study design and treatment

This trial was part of a multicentre, open-label, non-randomised, Phase 1 study separated into six parts (A, B, B2, C1, C2, and C3) (Fig. S1). The focus of this manuscript is part B: berzosertib in combination with cisplatin; the other parts will be reported elsewhere. Part B was a single-arm, 3 + 3 dose-escalation Phase 1 study evaluating the safety, tolerability and PK of berzosertib in combination with cisplatin in patients with advanced solid tumours. The study was conducted across two sites in the UK and three sites in the USA between July 2014 (first patient enrolled) and April 2017 (last patient discontinued treatment).

The starting dose of berzosertib was based on the emerging safety data from the ongoing part A study.^14^ Upon initiation of the part B study reported here, patients received berzosertib at what was then the highest dose tolerated in part A (or up to one dose level below). Following screening and baseline evaluations, patients received berzosertib i.v. (90 mg/m^2^; days 2 and 9) and cisplatin (40 mg/m^2^; day 1), administered in 21-day cycles. Based on safety data from two concurrent studies,^14,15^ a 7- to 14-day lead-in period with single doses of berzosertib (140 mg/m^2^) alone was also planned.

For the initial dose escalation, the dose of cisplatin was held constant, while the dose of berzosertib was escalated by a maximum of 50% in each cohort. After the berzosertib dose was escalated, the dose of cisplatin could be increased in order to find the maximum-tolerated dose (MTD) of berzosertib in combination with cisplatin; the MTD was defined as the highest dose of berzosertib tolerated in combination with a cisplatin dose between 60 and 100 mg/m^2^, inclusive. Safety data obtained through day 21 of cycle 1, including treatment-emergent adverse events (TEAE), serious TEAEs, laboratory values, electrocardiogram (ECG) results, and available PK data, were analysed to determine the next dose level. Patients received berzosertib with cisplatin until progressive disease (PD) or unacceptable toxicity. Patients who did not experience PD after cycle 4 were eligible to receive additional treatment cycles.

### Patients

Eligible patients were adults aged ≥18 years with histologically or cytologically confirmed advanced solid tumours that were metastatic or unresectable and for which standard curative or palliative measures did not exist or were no longer effective, or for whom regimens containing cisplatin might be considered. Eligible patients had a World Health Organization performance status 0 or 1; measurable disease defined by Response Evaluation Criteria in Solid Tumours (RECIST) version 1.1;^16^ adequate bone marrow, liver, and kidney function; and life expectancy of ≥12 weeks.

Key exclusion criteria included radiotherapy (except palliative), endocrine therapy, immunotherapy or chemotherapy within the 4 weeks prior to receiving study therapy; >6 cycles of prior treatment with cisplatin; ongoing toxicity or recent major surgery (≤2 weeks of first dose of study drug); active central nervous system (CNS) disease or symptoms within 4 weeks prior to treatment; cardiac conditions within 6 months prior to treatment; prior bone marrow transplant or radiation to >15% of bone marrow; and receiving medications that are known to be strong inhibitors or inducers of CYP3A4 that could not be discontinued at least 1 week before the start of treatment and for the duration of the study.

Full inclusion and exclusion criteria are shown in the Supplementary information.

### Study assessments and endpoints

The primary objective of the study was to assess the safety and tolerability of multiple ascending doses of i.v. berzosertib in combination with cisplatin, in patients with advanced solid tumours. The secondary objectives were to determine the MTD, PK, and preliminary anti-tumour activity of berzosertib in combination with cisplatin.

Safety evaluations (primary endpoint) included continuous assessment of TEAEs, which were graded using National Cancer Institute Common Terminology Criteria for Adverse Events version 4.0. TEAEs were assessed from the first dose to 14 days (±7 days) after the last dose of study therapy, while specific dose-limiting toxicity (DLT) events considered related/possibly related to the study drug were recorded up until the end of treatment cycle 1. Safety was evaluated throughout treatment and was used to inform dose-escalation decisions. DLTs were generally defined as any grade ≥3 haematologic or organ toxicity and any cardiac abnormality. Patients were eligible for DLT analysis if they had received all doses of berzosertib and cisplatin in cycle 1. The full definition of a DLT is shown in Supplementary information. The MTD was defined as the highest dose of berzosertib tolerated in combination with a cisplatin dose between 60 and 100 mg/m^2^, inclusive.

In cycle 1, plasma samples for PK analysis were collected both pre- and post-dose on days 2–5 (pre-dose, 0.5 h before the end of infusion, at the end of infusion, and 0.5, 1, 2, 3, 7, 23, 47, and 71 h after end of infusion) and day 9 (pre-dose). Plasma samples were also collected on day 2 of cycle 2 (pre-dose and 2 h after the end of infusion). Cumulative urine was also collected for PK assessments on days 2 and 3 of cycle 1 (pre-dose to 3, 3–7, 7–11, and 11–23 h). Berzosertib concentrations were quantified using a validated liquid chromatography tandem-mass spectrometry method and plasma PK were characterised by non-compartmental analyses using Phoenix WinNolin 6.4 (Certara USA Inc., Princeton, NJ, USA).

To assess tumour response, radiological restaging scans (computed tomography, magnetic resonance imaging, or bone scans) were performed at baseline, at the end of cycles 2 and 4, and at the end of every second or third cycle thereafter. Responses were assessed by the investigator according to RECIST version 1.1.

### Statistical analysis

Planned enrolment was ~30 patients. Sample size and power were based on a standard 3 + 3 dose-escalation rule using a binomial model. The maximally tolerated probability of toxicity associated with the dose selected by the standard 3 + 3 dose-escalation rule was calculated to range from ~17–26%, with an upper bound of 33%.

The safety set was defined as all patients who received at least one dose of study drug; patients within the safety set were further classified into those who received at least one dose of the study drug in the lead-in period (lead-in safety set) and those who received at least one dose during the combination period (combination safety set). PK data were collected in the PK analysis set, defined as all enrolled patients who received at least one dose of berzosertib and provided at least one measurable post-dose sample. For efficacy analyses, the full analysis set was used, comprising all enrolled patients who had received a baseline scan, at least one dose of study drug, and had at least one post-baseline disease assessment.

Standard non-compartmental methods were used to determine PK parameters. Continuous variables were summarised using descriptive summary statistics. Categorical variables (e.g. incidence of a TEAE) were summarised using frequency counts and percentages. TEAEs observed were summarised by system organ class and preferred term according to the Medical Dictionary for Regulatory Activities v.18.0 or higher.

## Results

### Patient demographics and disposition

Baseline and disease history characteristics of all 31 patients enrolled are presented in Table 1. The median age was 64.0 years (28–79); 45.2% were male (*n* = 14). The majority of patients had received prior platinum-based chemotherapy (*n* = 22).

**Table 1:** Patient demographics and baseline characteristics.

Characteristic	Total *N* = 31
Sex, *n* (%)	
Male	14 (45.2)
Female	17 (54.8)
Race, *n* (%)	
White	27 (87.1)
Black or African American	2 (6.5)
Asian	2 (6.5)
Median (range) age, years	64.0 (28–79)
<65 years	16 (51.6%)
≥65 years	15 (48.4%)
WHO PS, *n* (%)	
0	9 (29.0)
1	22 (71.0)
Prior chemotherapy, *n* (%)	31 (100.0)
Platinum-based chemotherapy^a^	22 (72.9)
Non-platinum-based chemotherapy	31 (100.0)
Primary tumour location (tumour type added where applicable), *n* (%)	
Colon/rectum (colorectal)	5 (16.2)
Breast	4 (12.9)
Other^b^	4 (12.9)
Ovary	4 (12.9)
Lung^c^	3 (9.7)
Pancreas	3 (9.7)
Bile duct (cholangiocarcinoma)	2 (6.5)
Eye (melanoma)	1 (3.2)
Oesophagus (squamous cell carcinoma)	1 (3.2)
Parotid gland (adenoid cystic carcinoma)	1 (3.2)
Prostate	1 (3.2)
Thymus (thymoma)	1 (3.2)
Uterus (endometrial)	1 (3.2)

*WHO PS* World Health Organization performance status.

^a^Ten patients received prior cisplatin.

^b^Included gastrointestinal stromal tumour, liposarcoma of inguinal region, squamous cell carcinoma, and unknown primary carcinoma.

^c^Included pleural mesothelioma, lung carcinoma, and non-small cell lung cancer.

**Fig. 1:**
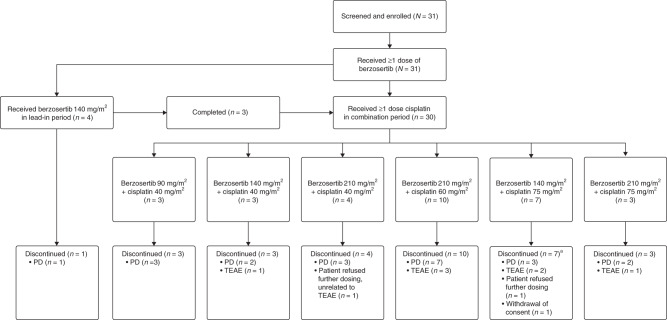
Patient disposition flow diagram in study part B. ^a^Included one patient who discontinued cisplatin due to a TEAE but continued treatment with single-agent berzosertib until disease progression. PD progressive disease, TEAE treatment-emergent adverse event.

Patient disposition is summarised in Fig. 1. All 31 patients received at least one dose of berzosertib. Part B initially included a 7- to 14-day lead-in period with berzosertib 140 mg/m^2^ monotherapy (*n* = 4). The lead-in period was later removed from the protocol based on available safety data from this study and a concurrent clinical study. Four patients received berzosertib 140 mg/m^2^ alone in the lead-in period for 15 days. One of these patients had PD during the lead-in period and therefore did not receive cisplatin. The other three patients who received berzosertib 140 mg/m^2^ monotherapy in the lead-in period went on to receive combination therapy.

In the combination period, the majority of patients discontinued treatment with berzosertib and cisplatin due to PD (21 patients [67.7%]). The median duration of treatment with berzosertib + cisplatin was 44 days (range 2–519).

### Dose escalation and DLTs

There were no DLTs during the monotherapy lead-in period. In the combination period, 30 patients were enrolled across the following six dose levels: berzosertib 90 mg/m^2^ in combination with cisplatin 40 mg/m^2^ (*n* = 3); berzosertib 140 mg/m^2^ in combination with cisplatin 40 mg/m^2^ (*n* = 3); berzosertib 210 mg/m^2^ in combination with cisplatin 40 mg/m^2^ (*n* = 4); berzosertib 210 mg/m^2^ in combination with cisplatin 60 mg/m^2^ (*n* = 10); berzosertib 140 mg/m^2^ in combination with cisplatin 75 mg/m^2^ (*n* = 7); and berzosertib 210 mg/m^2^ in combination with cisplatin 75 mg/m^2^ (*n* = 3) (Fig. 1).

Two DLTs occurred in two different patients while receiving the combination. One patient treated with berzosertib 210 mg/m^2^ in combination with cisplatin 60 mg/m^2^ experienced a DLT of grade 3 increase in ALT in the setting of hypotension and ischaemic colitis. The DLT was considered related to cisplatin and occurred on day 3 of cycle 1. The patient discontinued treatment and the DLT was resolved after ~2 weeks following treatment with antibiotics and mucolytic agents. The other DLT (grade 3 hypersensitivity reaction) was reported in a patient who received berzosertib 140 mg/m^2^ in combination with cisplatin 75 mg/m^2^. Approximately 20 min into the infusion of berzosertib, the patient became unresponsive for a brief period, developed an erythematous rash (grade 2) on her chest, and had 1 episode of vomiting (grade 1). The DLT was considered related to berzosertib and occurred on day 9 of cycle 1. The patient discontinued treatment and the DLT was resolved within 24 h following treatment with a steroid and an antihistamine.

Dose escalation was halted after completing the berzosertib 210 mg/m^2^ in combination with cisplatin 75 mg/m^2^ dose level due to chronic haematological toxicities that led to delays in cisplatin dosing. At this dose level, two patients experienced neutropenia (one grade 2 and one grade 3). As there was preclinical and clinical evidence of robust target engagement at lower doses,^15^ and as there was good tolerability in the absence of delays in cisplatin dosing, it was decided that berzosertib 140 mg/m^2^ (days 2 and 9) and cisplatin 75 mg/m^2^ (day 1) every 3 weeks (Q3W) would be the recommended Phase 2 dose (RP2D).

### Safety

The safety analysis set included 31 patients who received at least one dose of berzosertib, 30 of whom received at least one dose of both berzosertib and cisplatin. An overall summary of TEAEs for berzosertib in combination with cisplatin is shown in Table 2.

**Table 2:** Overview of TEAEs and TEAEs occurring in >15% of patients by preferred term (combination safety set).

Patients, *n* (%)	Berzosertib + cisplatin (all doses), *N* = 30	
	Any grade	Grade ≥ 3
TEAEs		
AEs	29 (96.7)	21 (70.0)
Serious AEs	11 (36.7)	9 (30.0)
Treatment-related AEs		
AEs	28 (93.3)	12 (40.0)
Serious AEs	3 (10.0)	1 (3.3)
AEs leading to study drug discontinuation	8 (26.7)	4 (13.3)
DLTs		2 (6.7)
TEAEs occurring in ≥15% of patients	Any grade	Grade 3–4^a^
Fatigue	17 (56.7)	0
Anaemia	15 (50.0)	5 (16.7)
Nausea	15 (50.0)	2 (6.7)
Constipation	11 (36.7)	0
Neutropenia	10 (33.3)	6 (20.0)
Vomiting	9 (30.0)	0
Abdominal pain	7 (23.3)	1 (3.3)
Diarrhoea	7 (23.3)	0
Hyponatraemia	7 (23.4)	2 (6.7)
Decreased appetite	6 (20.0)	0
Headache	6 (20.0)	1 (3.3)
Tinnitus	6 (20.0)	0
ALT increased	5 (16.7)	2 (6.7)
AST increased	5 (16.7)	1 (3.3)
Dizziness	5 (16.7)	0
Flushing	5 (16.7)	0
Hypokalaemia	5 (16.7)	1 (3.3)
Hypotension	4 (16.7)	1 (3.3)

*AE* adverse event, *ALT* alanine aminotransferase, *AST* aspartate aminotransferase, *DLT* dose-limiting toxicity, *TEAE* treatment-emergent adverse event.

^a^No grade 5 TEAE occurred.

Of the patients receiving combination therapy, 29 (96.7%) had at least one TEAE and 21 (70.0%) patients had at least one grade ≥3 TEAE (Table 2). The most common grade ≥3 TEAEs were neutropenia (six patients [20.0%]) and anaemia (five patients [16.7%]). The most common treatment-related AE of grade ≥3 was neutropenia, which occurred in six (20.0%) patients.

A total of 11 (36.7%) patients had at least one serious TEAE. The most common serious TEAEs were infusion-related reactions, and increases in alanine aminotransferase (ALT) and aspartate aminotransferase (AST), all of which occurred in two patients each. Furthermore, eight (26.7%) had at least one AE leading to treatment discontinuation, including the two patients who experienced serious infusion-related reactions (one related to berzosertib and scored as a DLT, and one related to cisplatin). The patient who discontinued treatment with cisplatin due to a cisplatin-related infusion reaction (hypotension and hypoxia) occurring on day 1 of cycle 13 continued berzosertib monotherapy up to cycle 28. There were no TEAEs that led to death during the treatment period. The majority of reported deaths were a result of disease-related complications or unconfirmed progression in the palliative care setting.

Finally, there were no clinically meaningful trends attributable to berzosertib treatment identified from laboratory results (serum chemistry, haematology, or urinalysis), vital signs, or ECG parameters.

### Pharmacokinetics

**Fig. 2:**
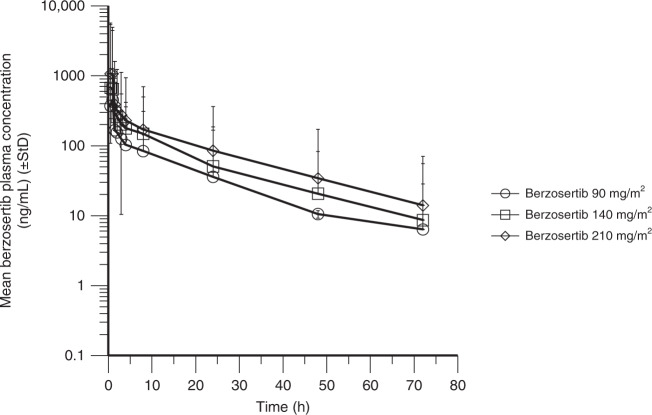
Plasma concentration–time profile for berzosertib in combination with cisplatin. StD standard deviation.

PK assessments for berzosertib were conducted during the monotherapy lead-in period, up to 3 days post administration, days 2–5 and 9 of cycle 1 and day 2 of cycle 2. Plasma PK parameters were determined for the four patients who participated in the monotherapy lead-in period (Table 3). A single data point in the terminal phase of one patient was excluded from PK parameter calculations as it was deemed implausible (10-fold increase from the previous concentration) and strongly skewed the results from the monotherapy lead-in subgroup. In combination with cisplatin, the PK characteristics of berzosertib were determined in 27 patients who received berzosertib 90–210 mg/m^2^, with profiles shown in Fig. 2 and PK parameters shown in Table 3. The PK characteristics of berzosertib in combination with cisplatin were consistent with the PK of corresponding doses of berzosertib monotherapy determined in part A of this study.^14^ The mean terminal elimination half-life of berzosertib monotherapy was determined in four patients to be ~17 h. Berzosertib plasma exposure was approximately dose proportional, based on the mean plasma concentrations and maximum observed plasma concentration values. The mean renal clearance was 4.7 L/h and the mean percentage of berzosertib excreted in the urine was 6%, indicating that renal clearance constitutes a minor mechanism of berzosertib clearance from the body.

**Table 3:** Mean (%CV) PK parameters of berzosertib in plasma in the lead-in period and for ascending doses of berzosertib in combination with cisplatin (PK analysis set).

Dose (mg/m^2^)	*C*_max_ (ng/mL)	AUC_0–∞_ (ng⋅h/mL)	*V*_ss_ (L)	CL (L/h)	*T*_1/2_ (h)
Lead-in period (berzosertib alone)					
140 (*n* = 4)	652 (28)	5670 (54)	1270 (34)	53.9 (40)	20.3 (22)
Cycle 1, day 2 (berzosertib and cisplatin)					
90 (*n* = 2)	458 (13)	2820 (15)	1260 (38)	61.1 (20)	17.0 (26)
140 (*n* = 8)	854 (63)	4870 (28)	1060 (36)	54.7 (31)	17.5 (34)
210 (*n* = 17)	1230 (43)	6740 (32)	1270 (28)	62.6 (32)	17.3 (17)

AUC_0–**∞**_ area under the concentration–time curve from the time of dosing extrapolated to infinity, *CL* clearance, *C*_*max*_ maximum observed plasma concentration, *CV* coefficient of variation (in %), *T*_*1/2*_ terminal phase half-life, *V*_*ss*_ volume of distribution at steady state.

### Efficacy

**Fig. 3:**
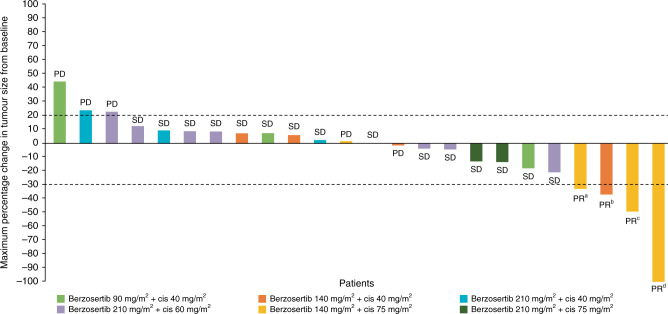
Maximum percent change in target tumour diameter from baseline. The dashed line at 20% represents PD, whereas the dashed line at −30% represents PR. Patients with PR had the following primary tumour types: ^a^high-grade serous ovarian cancer; ^b^pleural mesothelioma cancer; ^c^triple-negative breast cancer; ^d^castration-resistant prostate cancer. cis cisplatin, PD progressive disease, PR partial response, SD stable disease.

The full analysis set included 26 (83.9%) patients who received at least one dose of study drug, had a baseline scan, and had at least one post-baseline disease assessment. Four (15.4%) patients achieved PR (32.7–100.0% reduction in tumour lesion diameter) and with the duration of response ranging from 44 to 441 days. Fifteen (57.7%) patients achieved SD, one of which lasted for 172 days (Fig. 3).

All four patients who achieved PR, including those with advanced high-grade serous ovarian cancer (HGSOC), triple-negative breast cancer (TNBC), castration-resistant prostate cancer with neuroendocrine differentiation, and pleural mesothelioma, had been previously exposed to platinum-based regimens.

The patient with HGSOC was a 75-year-old *BRCA2* W2626Q carrier, who had undergone debulking, followed by i.v./intraperitoneal platinum and taxane-based treatment and subsequent carboplatin and doxorubicin, after which she was considered platinum-resistant. She then received paclitaxel and bevacizumab, as well as olaparib without response. She initiated treatment with berzosertib 140 mg/m^2^ and cisplatin 75 mg/m^2^. Cisplatin was reduced in cycle 2 to 60 mg/m^2^ due to grade 4 thrombocytopenia and grade 3 neutropenia during the first cycle. She achieved a confirmed PR and remained on trial for 14 weeks. The reason for treatment discontinuation was a rise in tumour markers, without documented RECIST progression.

The patient with TNBC was a 47-year-old female with a tumour harbouring TP53 R213* mutation and *RB1* deletion. After mastectomy, she received radiation and doxorubicin, cyclophosphamide, and paclitaxel in the adjuvant setting. She had recurrent disease after 17 months and was treated with re-excision, gemcitabine and cisplatin; she went on to experience PD after 3 months. She received berzosertib 140 mg/m^2^ in combination with cisplatin 75 mg/m^2^. She achieved an unconfirmed PR. After four treatment cycles, new brain and leptomeningeal progression were noted. Outside the CNS, progressive mediastinal adenopathy, and increases in pulmonary, bone, and liver lesions were also observed.

The patient with advanced castration-resistant prostate cancer with neuroendocrine differentiation was a 53-year-old man with a tumour harbouring duplication of exons 1–3 in *ATR*, as well as *BRCA2* and *TMPRSS2-ERG* somatic tumour mutations. He had previously received androgen deprivation therapy and platinum-based chemotherapy. Following treatment with berzosertib 140 mg/m^2^ and cisplatin 75 mg/m^2^, the patient achieved a confirmed PR lasting 69 weeks, with complete regression of liver metastases (Fig. 3).^17^ The patient later progressed, developing CNS metastases without evidence of disease progression elsewhere.

Finally, the patient with pleural mesothelioma was a 79-year-old male who had previously achieved SD as his best response following treatment with pemetrexed and cisplatin. He received five cycles of berzosertib 140 mg/m^2^ in combination with cisplatin 40 mg/m^2^ achieving PR, although this could not be confirmed in a subsequent CT scan because the patient discontinued due to grade 1 anorexia (possibly related) and grade 1 chronic kidney failure (unlikely related).

## Discussion

We evaluated the combination of berzosertib and cisplatin as part of the first-in-human trial of berzosertib. The sequential scheduling of chemotherapy with berzosertib was based on extensive preclinical data indicating that ATR inhibition has a maximal impact when administered 12–24 h after exposure to DNA-damaging agents. Importantly, the collective PK data, including comparison with part A of the study, suggest that pre-administration of cisplatin 24 h before berzosertib administration does not affect the PK profile of berzosertib.

In this portion of the study, the MTD of berzosertib in combination with cisplatin was not reached. Although no protocol-defined DLTs were reported at the highest doses evaluated (berzosertib 210 mg/m^2^ in combination with cisplatin 75 mg/m^2^), frequent episodes of neutropenia and leukopenia were observed, leading to delays in redosing of cisplatin. The RP2D was, therefore, determined as berzosertib 140 mg/m^2^ (days 2 and 9) in combination with cisplatin 75 mg/m^2^ (day 1) Q3W since this dose was generally well tolerated and did not lead to delays in cisplatin dosing. At the RP2D, the safety profile of the combination was generally consistent with that of cisplatin. Importantly, berzosertib exposure at 140 mg/m^2^ exceeded levels previously shown in preclinical models to result in robust target engagement. In in vivo mouse models, berzosertib 10–20 mg/kg has demonstrated efficacy in combination with chemotherapy,^15,18^ with the human equivalent dose estimated to be ~60 mg/m^2^.^19^ In addition, pharmacodynamic studies performed in the Phase 1 study of berzosertib with carboplatin demonstrated a reduction in serine 345-phosphorylated CHK1 (phospho-CHK1) at doses of berzosertib from 140 mg/m^2^.^15^ Therefore, it is likely that the combination achieved a biologically effective dose of berzosertib with full-dose cisplatin, without evidence of PK interactions.

One of the limitations of the current study could arguably be the lack of a comprehensive  pharmacodynamic sub-study that would have helped better understand the target engagement and durability of biological effects. It will be important to incorporate such translational evaluation in subsequent berzosertib studies. In addition to a reduction in phospho-CHK1, an increase in phospho-RAD50 has recently been shown to be a promising pharmacodynamic marker of ATR inhibition, resulting from a compensatory increase in ATM activity and therefore applicable in ATM-proficient cells.^20^ At later time points, prolonged γ-H2AX expression, compared with that achieved after cisplatin alone, may be useful to demonstrate the persistence of DNA damage afforded by the addition of ATR inhibition to chemotherapy.^21^

The majority of patients who received berzosertib in combination with cisplatin achieved disease control with PR or SD as the best response. Of note, PRs were observed in patients who had received prior platinum-based chemotherapy and had experienced disease progression. Notably, the patient with HGSOC and *BRCA2* mutation also did not benefit from treatment with olaparib. ATR inhibition can disrupt homologous recombination repair as well as DNA replication fork stability, two major mechanisms of PARP inhibitor resistance. These effects may have contributed to re-sensitising the tumour to cisplatin.^22^

The results of this study add to those of other already reported clinical trials that have evaluated the combination of berzosertib with different chemotherapeutic agents. In a Phase 1 study with berzosertib in combination with topotecan, three of five patients with platinum-resistant small cell lung cancer (SCLC) achieved PR or prolonged SD  lasting ≥6 months.^23^ A subsequent proof-of-concept Phase 2 study with berzosertib in combination with topotecan in patients with SCLC reported an objective response rate of 36% (9/25), with a median duration of response of 6.4 months, including those with platinum-resistant disease unlikely to respond to topotecan alone.^24^ Furthermore, in a randomised Phase 2 study, patients with platinum-resistant HGSOC, especially those who had a platinum-free interval <3 months, experienced longer PFS following treatment with berzosertib in combination with gemcitabine compared to gemcitabine alone.^25^ Taken together, these results may suggest that berzosertib, likely in combination with a synergistic chemotherapeutic drug, such as gemcitabine, a topoisomerase inhibitor or platinum compounds, may be clinically active in tumours under replicative stress, such as SCLC or HGSOC, and may potentially help overcome platinum and/or PARP inhibitor resistance.

As continued work with berzosertib ensues, it will be important to consider biomarker-driven approaches that target tumours harbouring *ATM* truncating mutations or ATM protein loss, *TP53* mutations, *BRCA1* or *BRCA2* mutations, or other alterations conferring homologous recombination repair deficiency. It will also be important to consider genomic changes that are likely to be associated with a high degree of replicative stress, such as *CCNE1* or *MYC* amplification, or *RB1* loss. Such tumours may be particularly susceptible to ATR inhibition and may ultimately define populations most likely to benefit from the addition of ATR inhibition to cisplatin or other DNA-damaging agents. The uniform *TP53* mutation in TNBC and the *TP53* mutation and *RB1* loss in SCLC have shaped the study design of the expansion phase of this study (parts C1, C2, and C3). Ultimately, the ability to combine biologically active doses of berzosertib with full doses of chemotherapy portends well for the continued leveraging of ATR inhibition to maximise cytotoxic responses.

In conclusion, berzosertib in combination with cisplatin was well tolerated and showed preliminary signs of efficacy. Further investigations, especially in the PARP inhibitor- and platinum-resistant setting, are warranted.

## Supplementary information

Supplementary information

## Data Availability

Any requests for data by qualified scientific and medical researchers for legitimate research purposes will be subject to the Merck KGaA, Darmstadt, Germany Data Sharing Policy. All requests should be submitted in writing to the Merck KGaA, Darmstadt, Germany data-sharing portal (https://www.merckgroup.com/en/research/our-approach-to-research-and-development/healthcare/clinicaltrials/commitment-responsible-data-sharing.html). When Merck KGaA, Darmstadt, Germany has a co-research, co-development, or co-marketing or co-promotion agreement, or when the product has been out-licensed, the responsibility for disclosure might be dependent on the agreement between parties. Under these circumstances, Merck KGaA, Darmstadt, Germany will endeavour to gain agreement to share data in response to requests.
