# Supplementary material for: Phase 1 study of the ATR inhibitor berzosertib in combination with cisplatin in patients with advanced solid tumours
Source: Br J Cancer. 2021 May 26;125(4):520–7. doi: 10.1038/s41416-021-01406-w (PMC8367944; doi:10.1038/s41416-021-01406-w)
Supplement: Supplementary file 1 — Supplementary information [file 41416_2021_1406_MOESM1_ESM.docx]

**Supplementary information**

## **Figure S1.** Study design.


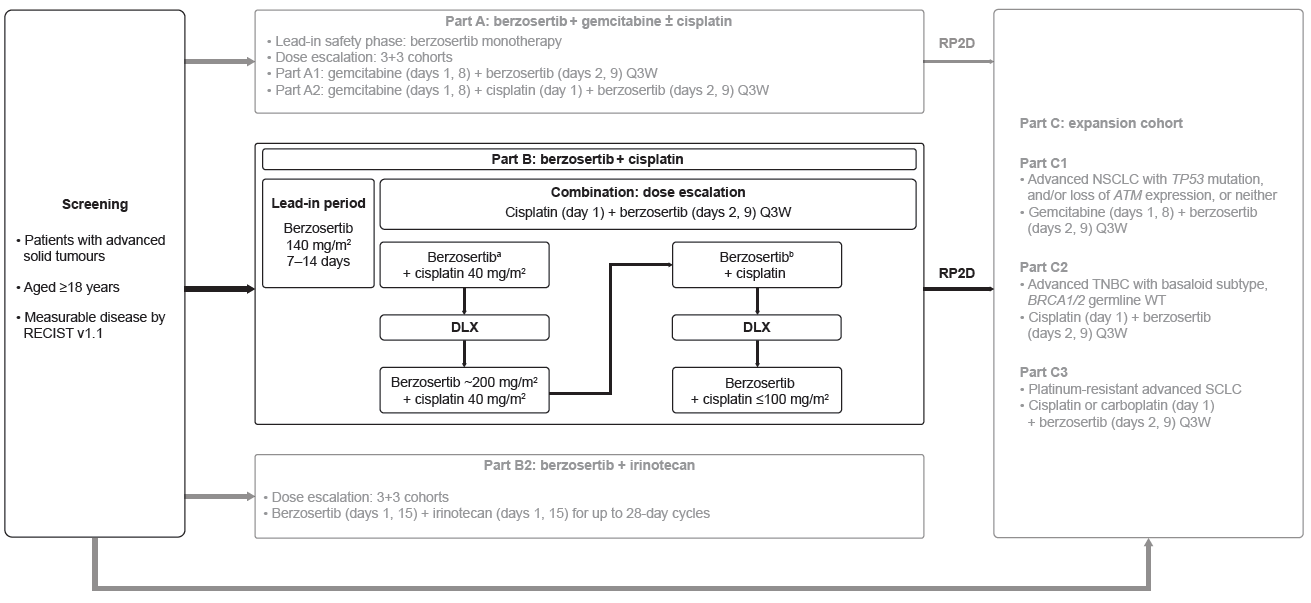


^a^The starting dose of berzosertib was based on the emerging safety data from the ongoing part A study. Upon the initiation of the part B study reported here, patients received berzosertib at what was then the highest dose tolerated in part A (or up to one dose level below).

^b^Dose of berzosertib kept constant while cisplatin dose was escalated.

## Abbreviations: DLX, dose level X; Q3W, every 3 weeks; RECIST, Response Evaluation Criteria in Solid Tumors; RP2D, recommended phase 2 dose.

## **Full inclusion and exclusion criteria**

### Inclusion criteria

- Male and female patients ≥18 years of age.
- Patients with histologically or cytologically confirmed advanced solid tumour that was metastatic or unresectable and for which standard curative or palliative measures did not exist or were no longer effective, or for whom regimens containing cisplatin might be considered, and with measurable disease according to Response Evaluation Criteria in Solid Tumors criteria (v1.1).
- World Health Organization performance status of 0 or 1.
- Life expectancy of ≥12 weeks.
- Haematological and biochemical indices within the ranges shown below at screening, with no clinically significant change in these values confirmed on the first day of dosing, before study drug administration:
  - Haemoglobin ≥8.0 g/dL
  - Absolute neutrophil count ≥1.5 x 10^9^/L
  - Platelet count ≥100 x 10^9^/L
  - Serum bilirubin ≤1.5 x upper limit of normal (ULN), unless the subject had known or suspected Gilbert’s syndrome
  - Alanine aminotransferase (ALT) and aspartate aminotransferase (AST) ≤2.5 x ULN or ≤5 x ULN in presence of liver metastases
  - Estimated glomerular filtration rate ≥60 mL/min
  - Prothrombin time <1.5 x ULN.
- Signed written, informed consent form and willing and able to comply with scheduled visits, treatment plan, lifestyle, laboratory tests, contraceptive guidelines, and other study procedures.

### Exclusion criteria

- Radiotherapy (except for palliative reasons), endocrine therapy, immunotherapy, or chemotherapy during the previous 4 weeks (6 weeks for nitrosoureas and Mitomycin-C, and 4 weeks for investigational medicinal products) or less than four drug half-lives, whichever is greater, before first dose of study drug.
- Prior chemotherapy
  - Greater than six cycles of prior treatment with cisplatin, unless discussed with and approved by the Vertex Pharmaceuticals (Vertex) medical monitor
    - History of prior dose reductions or dose interruptions while receiving cisplatin due to platinum-toxicity or intolerance to cisplatin, unless discussed with and approved by the Vertex medical monitor
  - Known history of grade 4 thrombocytopenia or grade 4 neutropenia while receiving prior therapy, unless discussed with and approved by the Vertex medical monitor.
- Unresolved toxicity of the National Cancer Institute (NCI) Common Terminology Criteria for Adverse Events (CTCAE) grade 2 or greater from previous anticancer therapy or radiotherapy, excluding:
  - Alopecia
  - Anaemia or leukopenia, as long as screening haemoglobin and absolute neutrophil counts fell within limits specified in inclusion criteria above
  - Other toxicities that, in the opinion of the investigator and the sponsor, should not exclude the patient.
- History of spinal cord compression or brain metastases, unless asymptomatic, treated, stable, and not requiring treatment with steroids for at least 4 weeks before first dose of study drug. Any history of leptomeningeal metastases.
- Female patients who were already pregnant or lactating or planned to become pregnant within 6 months of the last dose of study drug were excluded. Female subjects of childbearing potential were required to adhere to contraception guidelines as outlined in the protocol. Female patients were considered to be of non-childbearing potential if they underwent surgical hysterectomy or bilateral oophorectomy or were amenorrhoeic for over 2 years with a screening serum follicle-stimulating hormone level within the laboratory’s reference range for postmenopausal females.
- Pregnancy or lactation (female patients only), or male/female patients of reproductive potential not willing or able to employ a highly effective method of birth control/contraception to prevent pregnancy from screening until 6 months after receiving the last dose of study drug.
- Major surgery ≤2 weeks before starting study drug, or incomplete recovery from a prior major surgical procedure.
- Cardiac conditions as follows:
  - Clinically significant cardiovascular event within 6 months before study entry to include:
    - Congestive heart failure requiring therapy
    - Unstable angina pectoris
    - Myocardial infarction
    - Class II/III/IV cardiac disease (New York Heart Association)
    - Presence of severe valvular heart disease
    - Presence of a ventricular arrhythmia requiring treatment.
  - History of arrhythmia that is symptomatic or requires treatment (NCI CTCAE [version 3.0]), symptomatic or uncontrolled atrial fibrillation despite treatment, or asymptomatic sustained ventricular tachycardia. Subjects with atrial fibrillation controlled by medication were permitted.
  - Uncontrolled hypertension (blood pressure ≥160/100 despite optimal therapy)
  - Second- or third-degree heart block with or without symptoms
  - QTc >450 msec (by Fridericia’s correction) not due to electrolyte abnormality and that did not resolve with correction of electrolytes
  - History of congenital long QT syndrome
  - History of torsades de pointes (or any concurrent medication with a known risk of inducing torsades de pointes)
  - Clinically significant abnormality, including ejaculation fraction below normal institutional limits, present on transthoracic echocardiogram performed at screening.
- Prior bone marrow transplant or extensive radiotherapy to greater than 15% of bone marrow.
- Participation or plan of participation in another interventional clinical study while taking part in this phase 1 study of berzosertib. Participation in an observational study was acceptable.
- Any other condition that, in the investigator’s opinion, did not make the subject a good candidate for the clinical study, including:
  - History of human immunodeficiency virus 1 (HIV-1), HIV-2, hepatitis C virus, or unresolved hepatitis B infection
  - High medical risk because of non-malignant systemic disease including active uncontrolled infection
  - History of serious drug allergy or auto-immune disease
  - Diagnosis of Li-Fraumeni Syndrome or ataxia telangiectasia.
- Subject was the investigator or a sub-investigator, research assistant, pharmacist, study coordinator, other staff, or a relative of study personnel directly involved with the conduct of the study.
- Current therapy:
  - Patients receiving treatment with medications that are known to be strong inhibitors or inducers of CYP3A4 that could not be discontinued at least a week before start of treatment and for the duration of the study
  - Patients receiving treatment with ototoxic or nephrotoxic medications that could not be discontinued at least 7 days before first dose of study drug and for the duration of the study. Inadvertent or short-term use on study did not cause a subject to be ineligible. If a short course of therapy with nephrotoxic or ototoxic medication was anticipated and required, cisplatin was discontinued until 7 days after this course was completed.

## **Definition of dose-limiting toxicity (DLT)**

DLTs were identified throughout the dosing cycles and defined using NCI CTCAE (version 4.0). A DLT was defined as any of the following events that were considered related or possibly related to study drug:

- Neutropenia grade 4 for >7 days’ duration.

*Note*: In the event of a grade 4 neutropenia, a full blood count was performed no more than 7 days after the onset of the event to determine if a DLT occurred. The patient was monitored closely until resolution to grade 3 or less.

- Febrile neutropenia (fever of unknown origin without clinically or microbiologically documented infection).
- Infection (documented clinically or microbiologically) with grade 3 or 4 neutropenia (absolute neutrophil count <1.0 × 10^9^/L).
- Thrombocytopenia grade 3: associated with clinically significant bleeding; requiring platelet transfusion.
- Thrombocytopenia grade 4.
- Grade 3 or 4 toxicity to organs other than the bone marrow including grade 3 and 4 biochemical adverse events (AEs) and DLTs, excluding the following: grade 3 nausea; grade 3 vomiting in patients who had not received optimal treatment with anti-emetics; grade 3 diarrhoea in patients who had not received optimal treatment with antidiarrhoeal; any grade 3 elevation of AST, ALT, alkaline phosphatase (ALP) (of liver origin), or gamma-glutamyl transferase (GGT) lasting ≤7 days. Elevations in ALP were confirmed to be of liver origin by fractionation of ALP subsets.

*Note*: In the event of a grade 3 or higher elevation in ALT or AST, follow-up laboratory assessments were performed every 48 to 72 hours until reduced to grade 2 or less.

- A concurrent elevation of ALT >3 × the ULN and total bilirubin >2 × ULN in a patient in whom there was no evidence of biliary obstruction or other causes that could reasonably explain the concurrent elevation.
- Death due to drug-related complications.
- Cardiac: QTc prolongation (any QTc interval ≥500 msec or any change in QTc interval ≥60 msec from baseline) on ECG, unless related to an electrolyte abnormality and prolongation resolved with correction of electrolyte abnormality; any of the following (CTCAE criteria): grade 2 or greater ventricular arrhythmia (second or third degree AV block), severe sustained/symptomatic sinus bradycardia less than 45 beats per minute (bpm) or sinus tachycardia >120 bpm not due to other causes (e.g., fever), persistent supraventricular arrhythmia (e.g., uncontrolled/new atrial fibrillation, flutter, atrioventricular nodal tachycardia, etc.) lasting more than 24 hours, ventricular tachycardia defined as >9 beats in a row or any length of torsades de pointes (polymorphic ventricular tachycardia with long QTc), or unexplained recurrent syncope; symptoms suggestive of congestive heart failure with confirmed EF <40% (by 2D-echocardiogram or Multiple Gated Acquisition [MUGA] scan) or a relative decrease >20% from historical assessment of EF performed within 12 months; Troponin T: level that was consistent with myocardial infarction.
- Acute hypersensitivity has been recognised as an idiosyncratic reaction without clear relationship to dose of berzosertib occurring in <5% of patients on second infusion of berzosertib, and was not considered a DLT, regardless of grade or need for interruption or discontinuation.
- Any drug-related toxicity that caused interruption of treatment for >2 weeks (14 successive days). If a patient was deemed fit to restart treatment on Day 15 then this was not a DLT.

*Note*: If any change was made to the grade or causality of an AE during the study that could alter its DLT status, the sponsor was informed immediately as this could affect dose escalation decisions.
